# Perioperative medication therapy for Muslim patients in Germany undergoing oncological surgery: a retrospective study

**DOI:** 10.1186/s12910-024-01114-z

**Published:** 2024-10-18

**Authors:** Aysun Tekbaş, M. von Lilienfeld-Toal, F. Sayrafi, U. Settmacher

**Affiliations:** 1 Department of General, Visceral and Vascular Surgery, Jena University Hospital, Friedrich Schiller University Jena, Am Klinikum 1, 07747 Jena, Germany; 2 Research Programme “Clinician Scientist Programme”, Interdisciplinary Center of Clinical Research, Medical Faculty Jena, Jena University Hospital, Friedrich Schiller University Jena, Salvador-Allende-Platz 29, 07747 Jena, Germany; 3 Institute for Diversity Medicine, Ruhr-University Bochum, Universitaetsstr. 105, 44789 Bochum, Germany

**Keywords:** Muslim patients, Oncologic surgery, Intercultural medicine

## Abstract

**Purpose:**

Engagement of healthcare professionals with patients from diverse cultural and religious backgrounds is crucial in our multicultural society, where miscommunication and errors in medical history taking can lead to incorrect treatment. In particular, Muslim patients may present unique considerations due to their specific cultural and religious beliefs, which can significantly impact treatment outcomes. This study focuses on perioperative medication therapy for patients undergoing upper and lower gastrointestinal tract and pancreatic tumor surgery, specifically examining whether Islamic beliefs were duly considered in medication selection compared to a matching patient cohort.

**Materials and methods:**

Data from January 2004 to July 2023 were analyzed. Muslim patients were identified using the onomastic method and matched with non-Muslim patients at a 1:3 ratio based on age, gender, and procedure. Analysis included examination of subcutaneous, oral, and intravenous medications, with attention to ingredients and compatibility with Islamic principles.

**Results:**

Among 5272 patients, only 5 met the study’s inclusion criteria as Muslim patients, undergoing procedures such as anterior rectum resection, gastrectomy, and pancreatic head resection. Their religious affiliations were not documented in the admission records. According to the matched-pair analysis, consistent treatment was performed regardless of religious beliefs. All patients received subcutaneous medication, primarily enoxaparin, instead of fondaparinux, an Islam-compliant alternative. Intravenous heparin was used once for short period. Contrary to Islamic dietary restrictions and the availability of alternatives, capsules containing animal-derived gelatin and other non-compliant medications were administered orally.

**Conclusion:**

This study underscores the importance of acknowledging Muslim patients’ cultural and religious backgrounds in the perioperative setting, as failure to do so may lead healthcare professionals to overlook their potential alternative medication needs, which are essential for providing tailored medical care in modern societies. Integration of diversity-related topics into medical curricula is essential for better preparing physicians for clinical practice and ensuring patient-centered care.

## Introduction

In our multicultural and pluralistic societies, it is increasingly vital for healthcare professsionals to engage with a range of cultural and religious beliefs, with Muslim patients being one example, as these intersect with health in complex ways. According to the 2021 study “Muslimisches Leben in Deutschland 2020” conducted by the Federal Office for Migration and Refugees (BAMF), it was estimated that between 5.3 and 5.6 million Muslims were residing in Germany at that time, accounting for 6.4 to 6.7% of the total population in Germany [1]. Interestingly, the number of Muslims living in Europe is independent of migration caused by political or economic crises. According to the report “Europe’s Growing Muslim Population” by the Pew Research Center, which examines the future development of the Muslim population in Europe, significant growth of the Muslim population is expected, even if no further migration takes place. This is due to the younger age profile and higher fertility rates among Muslims compared to the non-Muslim population [2]. As a result of these demographic developments, healthcare professionals increasingly need to consider the specific needs of Muslim patients.

Effective communication is crucial, as unfamiliar cultural and religious practices can lead to misunderstandings, resulting in inadequate medical history taking and consequently incorrect treatment decisions [3, 4]. This can also lead to a violation of the patient’s potential preferences. Furtermore, cultural and spiritual beliefs play a significant role in treatment outcomes [5].

Therefore, particularly the care of Muslim cancer patients can be complex and may require thoughtful interdisciplinary collaboration [6].

These aspects make comprehensive cultural knowledge essential to ensure personalized therapy. Especially in the field of oncologic surgery for Muslim patients, there is a scarcity of information. Some sources provide insights into the cultural and religious considerations that may impact the care of Muslim patients undergoing surgery. The systematic review from Iqbal et al. has brought to light the distinct needs of Muslim individuals facing ostomies, emphasizing the significance of tailored support to address their specific concerns [7]. According to Iqbal et al., Muslim patients requiring ostomies should receive preoperative counseling from both surgeons and ostomy nurses. Importantly, these discussions should also involve faith leaders and/or hospital chaplains [7]. This holistic approach ensures that the emotional, spiritual, and religious aspects of the patient’s journey are considered and addressed. Kuzu et al. assessed the quality of life in Muslim patients after surgery for rectal carcinoma [8]. Two fundamental aspects of religious worship, namely praying and fasting, were significantly affected in Muslim patients who had a stoma due to sphincter-sacrificing surgery. To enhance the quality of life for Muslim patients with stomas, the author suggests that discussions about religious issues related to the presence of a stoma should be integrated into various stages of care. This includes preoperative counseling, the informed consent process, and again consultations with local religious authorities [8]. Almarzouqi et al. delve into the ramifications of incorporating porcine-derived tissue-engineered products within surgical contexts. Their findings indicate that, particularly in instances of life-threatening conditions and severe diseases, the utilization of porcine-derived materials may be deemed acceptable when equivalent non-porcine alternatives are unavailable [9].Gender considerations may also influence the care of Muslim patients. Lubna et al. examined gender preferences for endoscopists within a cohort of Muslim patients in Pakistan. The study revealed that a majority of Muslims in Pakistan had gender preferences, with both female and male patients displaying a preference for an endoscopist of the same gender [10]. Furthermore, a review examining the experiences of Muslim oncology patients undergoing palliative and end-of-life care revealed that the care extended to Muslim patients falls below the expected standard of culturally competent care. It was observed that the requirements of Muslim patients nearing the end of life, along with the needs of their families, are not adequately met [11].

As for our hospital, which is the only university hospital in the German federal state of Thuringia, the issue of culturally sensitive care is also insufficiently addressed. While there are isolated efforts to incorporate these topics into teaching and training events, there is a lack of practical guidelines that can be applied to patients across the hospital. It is worth noting that Thuringia is one of the federal states with the lowest proportion of people with a migration background (below 14%), compared to other states where the proportion can reach 35% or more, according to the 2021 microcensus from the Federal Statistical Office [12]. Consequently, the issue is only gradually gaining attention.

In conclusion, although direct research on oncologic surgery for Muslim patients is limited, these sources underscore the significance of culturally sensitive care and emphasize the pressing need for further investigation in this area. Furthermore, the insufficient integration of diversity-related medical topics into the curriculum leaves physicians ill-equipped for clinical practice. Kronenthaler et al. elucidate how Muslim patients in Germany are often subject to perceptions in primary care settings that lack thoughtful consideration, resulting in stereotyping due to a deficiency in comprehending cultural and religious contexts [13]. Consequently, it becomes evident that both scientific exploration and curricular enhancement are imperative steps towards cultivating patient-centered, high-quality medical care in this specific domain.

The objective of our study is to shed light on the perioperative pharmacological therapy of Muslim patients undergoing oncological resection at our hospital, with the aim of drawing conclusions that can be applied to hospitals across Germany. It is widely recognized that for Muslims, alcoholic and animal-derived ingredients from non-halal-slaughtered animals, predators and pigs are prohibited. However, the principles of “dire necessity” and “transformation” in Islamic bioethics are key to evaluating whether exceptions to dietary prohibitions can be made in critical medical situations [14, 15]. The principle of “dire necessity” permits the use of otherwise prohibited substances if they are essential for preserving health or life, and no suitable alternative exists. Another important concept is “transformation” (tahaarah), which in Islamic law refers to the process by which an impure substance becomes pure through chemical or physical changes. This highlights that there is an intersection between Islamic bioethics and modern science, particularly regarding the ethical considerations around the use of porcine (pig-derived) and alcoholic products in medicine [14–17]. It is often not possible to make a generalized statement about such matters, as the ethical considerations vary by situation and by personal attitudes.

## Material & methods

### Study design

A retrospective data analysis was undertaken to examine the perioperative medication therapy of patients undergoing surgery for upper and lower gastrointestinal tract as well as pancreatic tumors of various entities at our department from January 2004 to July 2023.

### Data source

Patient data were retrieved from our electronic medical records system, SAP^®^ i.s.h.med^®^ (Systems, Applications, and Products in Data Processing, Berlin, Germany), utilizing relevant ICD-10 codes (International Statistical Classification of Diseases and Related Health Problems, 10th Revision) for upper and lower gastrointestinal tract and pancreatic resections due to tumors.

### Selection of muslim patients

To identify Muslim patients within the dataset, the onomastic method [18] was employed, enabling the selection of individuals with Muslim names according to Islamic naming conventions, common and historical names as well as cultural and regional variations.

### Examination of perioperative medication therapy

The perioperative medication therapy of the selected patients was rigorously scrutinized by extracting and analyzing data from their electronic patient records (COPRA6 RM1.0, COPRA System GmbH, Berlin, Germany). In this process, we conducted a thorough examination of the intravenous, oral and subcutaneous medications administered in the first 10 postoperative days after oncological surgery to determine their ingredients. We specifically assessed whether these medications contained any alcoholic and/or animal-derived substances and whether there were alternative medications available that would align with Islamic ethical principles.

### Matched pair analysis

Patients within a ± 5-year age range and of the same gender, undergoing comparable surgical procedures during the specified period, were matched at a 1:3 ratio with those in the study cohort. To assess whether significant differences exist in the number of administered medications on the respective days between the test cohort and the matched groups, generalized estimating equations with a negative binomial distribution were employed. Significance was attributed to p-values < 0.05.

## Results

**Fig. 1:**
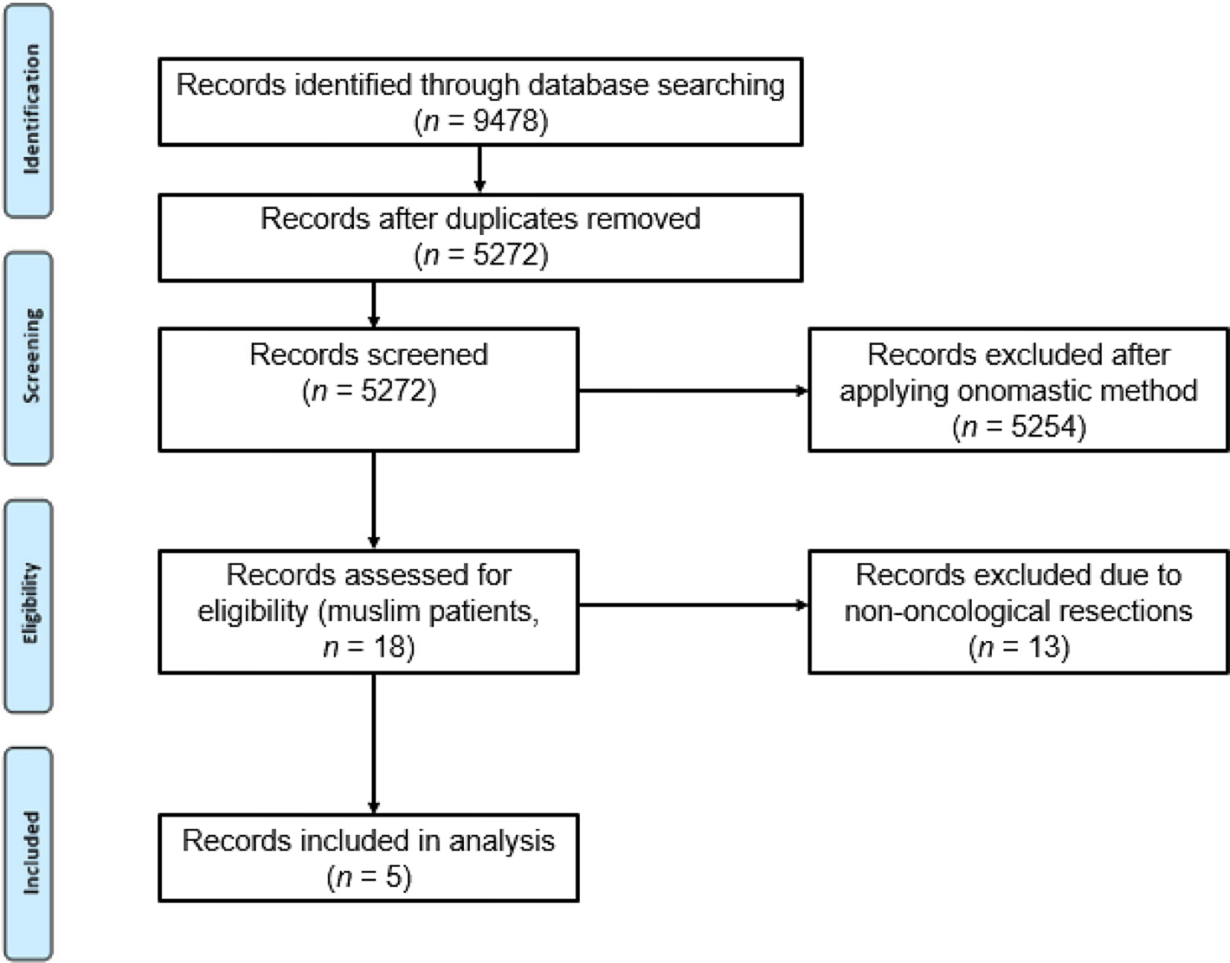
Process of identifying appropriate patient files from SAP^®^ i.s.h.med^®^ records

Out of a total of 5272 patients, only five met the inclusion criteria for our study (Fig. 1). Two patients underwent an anterior rectum resection, two a gastrectomy, and one a pancreatic head resection for either a primary tumor or metastasis, as detailed in Table 1. The mean length of postoperative hospital stay was 18,6 days (± 5,2). There was no documentation of religious beliefs and dietary habits in the general admission documents of the patients, indicating a gap in the assessment of patients’ religious and cultural backgrounds.

**Table 1:** Analyzed patients’ sex, diagnosis and surgical procedure. The age is provided as age-range (P1 and P2: 40–50, P3: 50–60, P4 and P5: 60–70). P: patient, f: female, m: male, HIPEC: hyperthermic intraperitoneal chemotherapy

*P* ID	Sex	Diagnosis	Surgical procedure
1	f	Peritoneal metastasis of adenocarcinoma of the ovary T3c N1 (10/36) L1 V0 G3	Peritonectomy, anterior rectal resection, ileocecal resection, cholecystectomy, pelvic lymphadenectomy, HIPEC
2	m	Adenocarcinoma of the sigmoid colon T3 N0 (0/22) V1 L1 Pn0 M0 G3	Anterior rectal resection with primary anastomosis
3	f	Moderately differentiated adenocarcinoma of the stomach T0 N0 (0/15) L0 V0 M0	Gastrectomy with D2 lymphadenectomy, cholecystectomy
4	m	Poorly differentiated diffuse adenocarcinoma of the stomach T4a N2 (4/18) M1 (PER) L1 V0 Pn0 G3	Gastrectomy with D2 lymphadenectomy, right hemicolectomy, terminal ileostomy
5	m	Moderately differentiated adenocarcinoma of the distal bile duct T3 N0 (0/12) L0 V0 Pn1 M0 G2	Pancreatic head resection

The patients in the matched cohort (Table 2) were subject to similar operations, their mean length of postoperative hospital stay was also alike (14,6 days ± 6,4). In comparison to the study cohort, in the matched group, the religious affiliation was queried for nine patients (60%). One patient reported being protestant, and eight had no religion. Dietary habits were not mentioned.

**Table 2:** Sex, diagnosis and surgical procedure of matched patients. The age is provided as age-range (P1a-c and P2a-c: 40–50, P3a-c: 50–60, P4a-c and P5a-c: 60–70). P: patient, f: female, m: male

*P* ID	Sex	Diagnosis	Surgical procedure
1a	f	Adenocarcinoma of the rectum T2 N1b (2/12) L0 V0 M0	Laparoscopic low anterior resection of the rectum with protective ileostomy
1b	f	Moderately differentiated adenocarcinoma of the sigmoid colon T4a N1b (2/23) L1 G2 M1	Anterior rectal resection and partial peritonectomy
1c	f	Moderately differentiated adenocarcinoma of the rectum T2, N1a (1/24) V0 L0 Pn0 G2	Anterior rectal resection with protective ileostomy
2a	m	Mucinous adenocarcinoma of the rectum T3 N0 (0/19) L0 V0 G2	Rectum resection, cystectomy, bilateral ileal conduit, descendostomy
2b	m	Moderately differentiated adenocarcinoma of the rectum T2 N2b (8/38) L0 V0 Pn0 G2	Anterior rectal resection with protective ileostomy
2c	m	Moderately differentiated adenocarcinoma of the rectum T3 N2b (15/15) L1 V1 Pn0 M1b	Anterior rectal resection with protective ileostomy
3a	f	Adenocarcinoma of the gastroesophageal junction T3 N2 (3/62) L0 V1 Pn0	Transhiatal extended gastrectomy with esophagojejunostomy, D1 and D2 lymphadenectomy, simultaneous cholecystectomy
3b	f	Poorly differentiated signet-ring cell carcinoma of the stomach T3 N1 (2/35) L0 V0 Pn0	Laparoscopic gastrectomy with D1 and D2 lymphadenectomy, simultaneous cholecystectomy
3c	f	Signet-ring cell carcinoma of the stomach T3 N0 (040) L0 V0 Pn0 M0	Gastrectomy with D1 and D2 lymphadenectomy, simultaneous cholecystectomy
4a	m	Adenocarcinoma of the esophagogastric junction T2 N3b (16/28) L1 V0	Gastrectomy with removal of the distal esophagus, D2 and D3 lymphadenectomy
4b	m	Moderately differentiated adenocarcinoma of the stomach T3 N0 (0/26) L0 V0 G2	Transhiatal gastrectomy and cholecystectomy
4c	m	Adenocarcinoma of the gastric antrum T2 N0 (0/81) L0 V0 Pn0 M1	Gastrectomy and cholecystectomy
5a	m	Poorly differentiated ductal adenocarcinoma of the pancreas T2 N0 (0/14) L0 V0 Pn1 G3	Pancreatic head resection
5b	m	Differentiated ductal adenocarcinoma of the pankreas T2 N2 (7/28) L1 V0 Pn1 G3	Pancreatic head resection with short-segment resection of the superior mesenteric vein
5c	m	Adenocarcinoma of the pancreas T1c N0 (0/27) L0 V0 Pn1 cM1	Pancreatic head resection

Table 3 depicts the medications administered to the 20 patients on the 1st, 3rd, 5th, 7th and 10th postoperative days. All patients received subcutaneous anticoagulation, with enoxaparin being the primary choice. There were three singular instances where intravenous heparin was briefly administered. Enoxaparin, tinzaparin, and heparin, all fall under the category of low molecular weight anticoagulants, with their origins mostly traced back to porcine intestinal mucosa. However, there is a synthetic alternative (Table 4) available, the factor Xa inhibitor fondaparinux. Therefore, this might be preferred for Muslim patients.

**Table 3:** Medications administered on the 1st, 3rd, 5th, 7th, and 10th postoperative days that are not halal-compliant. There is uncertainty with medications marked in italics, as it is not clear solely from the package insert whether the respective ingredient is of plant or animal origin. Enoxa.: enoxaparin, PCC: prothrombin complex concentrate, ODT: orally disintegrating tablet, suppl.: supplement, cps.: capsule, UDCA: ursodeoxycholic acid, tinza: tinzaparin, Vit.: vitamin, *early discharge

*P* ID	Medication d1			Medication d3			Medication d5			Medication d7			Medication d10			Home medication
	iv	sc	oral	iv	sc	oral	iv	sc	oral	iv	sc	oral	iv	sc	oral	
1	*Propofol*	Enoxa	0	0	Enoxa	0	*Propofol*	Enoxa	0	*Propofol*, Heparin	0	0	Heparin	0	0	0
1a	0	Enoxa	*Quetiapine*	0	Enoxa	*Quetiapine*	0	Enoxa	Potassium cps., *Quetiapine*	0	Enoxa	Potassium cps., *Quetiapine*	0	Enoxa	Potassium cps., *Quetiapine*	*Quetiapine*
1b*	0	Enoxa	0	0	Enoxa	0	0	Enoxa	0	-	-	-	-	-	-	0
1c*	0	Enoxa	0	0	Enoxa	0	0	Enoxa	0	0	Enoxa	0	-	-	-	0
2	0	Enoxa	0	0	Enoxa	Simethicone cps.	0	Enoxa	0	0	Enoxa	0	0	Enoxa	Potassium cps.	0
2a	0	Enoxa	0	0	Enoxa	0	0	Enoxa	Potassium cps.	0	Enoxa	Potassium cps.	0	Enoxa	Potassium cps.	0
2b	0	Enoxa	0	0	Enoxa	0	0	Enoxa	0	0	Enoxa	0	0	Enoxa	0	0
2c	0	Enoxa	0	0	Enoxa	0	0	Enoxa	0	0	Enoxa	0	0	Enoxa	0	0
3	PCC	Enoxa	0	0	Enoxa	0	0	Enoxa	Lorazepam ODT	0	Enoxa	Lorazepam ODT, *Pipamperone syrup*	0	Enoxa	Lorazepam ODT, *Pipamperone syrup*	0
3a	0	Enoxa	0	0	Enoxa	0	0	Enoxa	0	0	Enoxa	0	0	Enoxa	0	0
3b	0	Enoxa	0	0	Enoxa	0	0	Enoxa	0	0	Enoxa	0	0	Enoxa	*Furosemide*	0
3c	0	Enoxa	0	0	Enoxa	0	0	Enoxa	0	0	Enoxa	0	0	Enoxa	0	0
4	0	Enoxa	Ferro sanol^®^ cps.	0	Enoxa	0 (Ferro sanol^®^ cps. paused)	0	Enoxa	0 (Ferro sanol^®^ cps. paused)	0	Enoxa	0 (Ferro sanol^®^ cps. paused)	0	Enoxa	0 (Ferro sanol^®^ cps. paused)	Ferro sanol^®^ cps.
4a	0	Enoxa	0	0	Enoxa	0	0	Enoxa	0	0	Enoxa	0	0	Enoxa	0	0
4b	0	Enoxa	Lorazepam ODT, *Torasemide*	0	Enoxa	Lorazepam ODT, *Torasemide*	0	Enoxa	*Torasemide*	0	Enoxa	*Torasemide*	0	Enoxa	*Torasemide*	Lorazepam ODT, *Torasemide*
4c	0	Enoxa	0	0	Enoxa	0	0	Enoxa	0	0	Enoxa	0	0	Enoxa	0	0
5	0	Tinza	0	0	Tinza	0	0	Tinza	0	0	Tinza	0	0	Tinza	UDCA cps., morphine sulfate cps.	0
5a	*Propofol*	0	Tacrolimus cps.	Heparin	0	Tacrolimus cps.	Heparin	0	Tacrolimus cps.	Heparin	0	Tacrolimus cps., Vit. D3 cps., Pancrelipase cps.	0	Heparin	Tacrolimus cps., Vit. D3 cps., Pancrelipase cps.	Tacrolimus cps., Vit. D3 cps.
5b	Heparin	0	0	Heparin	0	0	Heparin	0	0	0	Enoxa	Pancrelipase cps.	0	Enoxa	Pancrelipase cps., Simethicone cps.	0
5c	0	Tinza	0	0	Tinza	0	0	Tinza	0	0	Tinza	Pancrelipase cps.	0	Tinza	Pancrelipase cps.	0

**Table 4:** Possible alternatives to non-compliant medications. PCC: prothrombin complex concentrate, ODT: orally disintegrating tablet, cps.: capsule, UDCA: ursodeoxycholic acid, Vit.: vitamin

Administration route	Administered medication	Alternative
intravenous	**Heparin**	*Depending on indication*, *possibly subcutaneous Fondaparinux*
	**PCC**	-
	**Propofol**	*Depending on indication (clarification with the manufacturing company needed)*
subcutaneous	**Enoxaparin/Tinzaparin/Heparin**	Fondaparinux
oral	**Ferro sanol**^®^ cps.	Tardyferon
	**Furosemide/Torasemide**	Intravenous application *(clarification with the manufacturing company needed)*
	**Lorazepam ODT**	Lorazepam tablet
	**Morphine sulfate cps.**	Morphine sulfate orodispersible tablet
	**Pancrelipase cps.**	Nortase cps.
	**Pipamperone syrup**	*Depending on indication*,* other oral neuroleptics (clarification with the manufacturing company needed)*
	**Potassium cps.**	Potassium effervescent tablets
	**Quetiapine**	*Depending on indication (clarification with the manufacturing company needed)*
	**Simethicone cps.**	Simethicone tablet
	**Tacrolimus cps.**	*Depending on immunosuppressive regimen, possibly no alternative*
	**UDCA cps.**	UDCA tablet
	**Vit. D3 cps.**	Vit. D3 tablet

Prothrombin complex concentrate (PCC) and propofol were used as intravenous rescue medications. PCC often includes heparin due to the manufacturing process. In the case of propofol, the assessability regarding its compatibility with a “halal” (permissible) dietary approach is not certain from its ingredients, as the glycerol contained therein can be of animal, plant, or synthetic origin. The same applies to oral medication with pipamperone and quetiapine, which contain ingredients such as glycerol and polysorbate, respectively. Furosemide and torasemide both contain magnesium stearate, the origin of which (plant or animal derived) is also unclear.

For the remaining oral medications, the main issue lies in the processing of substances as capsules, which contain gelatin, a mixture of animal proteins. However, there are often alternatives for these medications, allowing them to be avoided in the case of Muslim patients (Table 4). Pancrelipase, which contains a combination of digestive enzymes derived from porcine pancreas, can be replaced by plant-based enzyme preparations (e.g., Nortase) for individuals following religious dietary practices or a vegetarian diet.

Overall, upon scrutinizing the significance of differences in the number of medications administered on corresponding days between the test cohort and the matched groups, it becomes apparent that all patients received proper and equitable treatment tailored to their individual circumstances (p values > 0.05, Table 5). However, there was a lack of attention to addressing cultural and religious considerations. Only on the 3rd postoperative day, there was significant less non-halal intravenous medication administered in the tested group (*p* = 0.024).

**Table 5:** Rate ratio with 95% CI and p-value between tested and matched cohort for administered number of non-halal medications on the 1st, 3rd, 5th, 7th, and 10th postoperative day. *Patient 1a 0 iv medication, 1b and 1c early discharge; rate ratio not determinable

Administration route	Medication day	Rate ratio (95% CI)	*p*-value
Intravenous	d1	3 (0.272–33.085)	0,370
	d3	< 0.001	0.024
	d5	1.5 (0.094–23.981)	0.774
	d7	5.6 (0.386–81.181)	0.207
	d10*	-	-
Subcutaneous	d1	1.154 (0.881–1.511)	0.298
	d3	1.154 (0.881–1.511)	0.298
	d5	1.154 (0.881–1.511)	0.298
	d7	0.862 (0.533–1.393)	0.543
	d10	0.8 (0.516–1.24)	0.318
Oral	d1	0.75 (0.226–2.491)	0.639
	d3	0.75 (0.075–7.468)	0.806
	d5	0.6 (0.065–5.51)	0.652
	d7	0.622 (0.06–6.423)	0.69
	d10	1.182 (0.448–3.117)	0.736

## Discussion

We conducted a retrospective analysis to evaluate perioperative medication management in Muslim patients who underwent surgery for upper and lower gastrointestinal, as well as pancreatic tumors. Covering the period from January 2004 to July 2023, this analysis compared these patients with a matched cohort of non-Muslim patients to assess how well culturally sensitive aspects were integrated into their medication treatment. Medications like enoxaparin, derived from porcine sources, were commonly used, although they may be unsuitable for Muslim patients given the availability of synthetic alternatives such as fondaparinux. This shows, that despite equitable medical treatment across patients in both the study and the matched cohorts, there was a notable lack of attention to religious and cultural considerations. The fact that, statistically, fewer non-halal medications were administered on the 3rd postoperative day does not appear to have been done intentionally. Interestingly, no documentation of religious beliefs or dietary habits was found in the study cohort, while 60% of the matched cohort had religious affiliations recorded. The electronic patient record in our clinic generally allows for the query of religious affiliation, with 41 different options available (e.g., including various sects of Christianity, Judaism, Islam, Buddhism, and Hinduism), as part of administrative procedures, but the query was evidently not carried out consistently. Actually, according to the German Patient Rights Act (Patientenrechtegesetz), patient privacy is a fundamental right, and personal details, including religious beliefs, are not routinely collected unless directly relevant to the patient’s care. On the other hand, in Germany, people are often stereotyped based on their appearance, as reported in the 2017 report “Discrimination Experiences in Germany” by the Federal Anti-Discrimination Agency [19]. Furthermore, the recent NaDiRa Report 2023 (National Discrimination and Racism Monitor) sheds crucial light on the prevalence of racism within the German healthcare system [20]. Titled “Racism and Its Symptoms,” the report reveals that discrimination and racism are pervasive issues, varying by social group, personal characteristics, and specific contexts. These problems are frequently encountered in healthcare settings, affecting not just those who are directly subjected to bias but also having broader implications for society [20]. Therefore, instead of making assumptions, stereotyping, or even engaging in racism based on appearance, direct communication and including questions about religious affiliation in the administration procession and/or medical history should be encouraged to address and incorporate any special needs into the therapy. Only in this way can doctors determine the patient’s perspective on the intake of certain medications and whether they have any personal concerns regarding this, irrespective of religious obligations. Finally, one cannot make a blanket statement about a person’s attitude based solely on their group affiliation as the ethical considerations can vary. Decision-making is complex and might be impacted by multiple factors with religion being one of those. When we turn our focus to the specific treatment of Muslim patients, it is important to emphasize that the primary challenge in the context of oncological surgery for these patients is not the surgery itself [7–9]. Although Islam generally encourages individuals to seek all available medical treatments for their recovery [21], there are complex aspects of Islamic jurisprudence that must be considered [16]. Primarily, Islamic jurisprudential guidelines suggest that patients should seek healthcare when experiencing symptoms that could potentially harm their well-being or when an illness impairs their ability to perform religious duties. Modern clinicians on the other hand focus on the medical aspects of healthcare decisions, emphasizing timely intervention based on clinical evidence and patient symptoms [16]. However, there is a need to adapt (perioperative) therapies, including medications, to meet the specific needs and beliefs of Muslim patients [22]. This includes considering the prohibition of alcohol and animal-derived ingredients in medications, provided suitable alternatives exist [22]. If no alternatives are available (e.g. PCC), Muslim patients are encouraged to accept also non-halal medications for the sake of their health [9, 21, 22]. Yet, this should be explained to the patient [22]. In general, both from an ethical and a purely medical perspective, actions should be based on preventing or removing harm (or potential for harm) from illness through medical treatment, and ensuring the efficacy of clinical treatment. These aspects underscore the necessity for dialogue between Islamic jurists and clinicians to bridge the gap between religious guidance and medical practice. Integrating religious considerations with medical advice can lead to more holistic patient care, ensuring that both ethical and clinical standards are met [16].Another aspect, based on our data, is that classifying medications as halal, vegan, or vegetarian itself is a challenging task. Neither the medical staff nor the patients are typically familiar with the detailed composition of the ingredients, nor are they able to assess whether these substances align with specific dietary or religious requirements. It is unreasonable to expect that either staff or patients should possess comprehensive knowledge of all the ingredients in medications. Consequently, physicians often remain unaware of the potential implications certain ingredients may have for Muslim patients or individuals with specific dietary preferences.

To address this issue, clear and transparent labeling by pharmaceutical manufacturers [17], indicating whether a medication is suitable for vegan, vegetarian, or halal consumption, could be highly beneficial. Currently, only a small number of companies provide explicit information regarding whether potentially questionable ingredients are plant-based or animal-derived (e.g. magnesium stearate). Furthermore, in our own ingredient analysis, it is possible that non-halal substances may have been inadvertently overlooked for the same reason. This lack of clarity and awareness complicates the ability of healthcare professionals to administer medications in a manner that respects the cultural and religious values of their patients.

Although the findings of this study provide valuable insights into the perioperative therapy of Muslim patients, there are also limitations and broader implications to consider. One limitation is the onomastic method used for patient selection [18]. While it enabled us to identify Muslim patients based on their names, it may not be entirely accurate. The mere presence of names cannot conclusively ascertain religious affiliation, as individuals bearing Muslim names may not necessarily adhere to Islamic beliefs and practices, while those with more conventional names may indeed follow Islamic principles. Thus, there might be a margin of error in our patient selection process. Furthermore, due to the small number of patients (*n* = 5) and the fact, that this is a single-center study, the data is limited in terms of significance. It is worth noting that ICD diagnoses may not always accurately reflect the true number of potentially oncologically resected patients because misdocumentation can occur. This could lead to an underestimation of the total patient population undergoing oncological surgery. Consequently, the percentage of Muslim patients among these cases may be higher than initially presumed.

In conclusion, incorporating the findings of this research into clinical practice is crucial. It can pave the way for a more patient-centered and culturally sensitive approach in surgery specifically and medicine in general. Acknowledging and accommodating unique needs of patients can improve the overall quality of care and enhance the well-being of the patients, aligning with the principles of ethical and inclusive healthcare.

## Conclusion

These findings reveal that healthcare professionals in Germany are not sufficiently sensitized to the potential alternative medications needs of Muslim patients. They highlight the need for increased awareness and consideration of the cultural and religious backgrounds of Muslim patients in the perioperative setting because tailoring medication choices to align with the respective belief can contribute to more patient-centered and culturally sensitive care in the context of oncologic surgery. A possible way forward to address these issues is the integration of diversity-related topics into the medical curriculum.

## Data Availability

The datasets analyzed during the current study are available on request from the corresponding author.

## Abbreviations

BAMF: Federal Office for Migration and Refugees
ICD-10: International Statistical Classification of Diseases and Related Health Problems, 10th Revision
SAP: Systems, Applications, and Products in Data Processing
PCC: Prothrombin Complex Concentrate
F: Female
M: Male
HIPEC: Hyperthermic Intraperitoneal Chemotherapy
Enoxa: Enoxaparin
e.g: Exempli gratia = for example
ODT: Orally Disintegrating Tablet
NaDiRa: National Discrimination and Racism Monitor
suppl: Supplement
Cps: Capsule
P: Patient
UDCA: Ursodeoxycholic Acid
Tinza: Tinzaparin
vit: Vitamin
